# Examining Measurement Invariance of Depression among Male and Female in Chinese Older Adults

**DOI:** 10.1093/geroni/igab046.3113

**Published:** 2021-12-17

**Authors:** Roberto Melipillán, Mengyao Hu

**Affiliations:** 1 Universidad del Desarrollo, Concepcion, Bio-Bio, Chile; 2 University of Michigan, Ann Arbor, Michigan, United States

## Abstract

Depression of older adults is an important public health concern. With the increasing popularity of cross-cultural research and comparison studies, researchers are facing a difficult problem: responses to the depression scales obtained from different population groups may not always be comparable. This study examines the measurement invariance of the 10-item version of the Center for Epidemiological Studies Depression (CES-D) Scale across male and female in Chinese older adults. Data are drawn from the baseline wave of the China Health and Retirement Survey (CHARLS), a national survey conducted biennially with a sample of the Chinese population who are 45 years of age or older. The final sample size includes 15,977 respondents; 53.2% of whom are female. The mean age for the sample is 58.3 (SD = 10.2). Measurement invariance (MI) tests based on Multiple Group Categorical Confirmatory Factor Analyses (MGCCFA) was performed. Results show that full scalar model was not supported, and question items invariant across groups were identified. These results indicate that any mean comparisons of CES-D across Chinese male and female older adults not accounting for the noninvariance in the items could be biased, highlighting the importance of performing MI tests before conducting mean comparisons across groups.

